# PET Imaging of Astrogliosis and Tau Facilitates Diagnosis of Parkinsonian Syndromes

**DOI:** 10.3389/fnagi.2019.00249

**Published:** 2019-09-11

**Authors:** Sonja Schönecker, Matthias Brendel, Carla Palleis, Leonie Beyer, Günter U. Höglinger, Elisabeth Schuh, Boris-Stephan Rauchmann, Julia Sauerbeck, Guido Rohrer, Stefan Sonnenfeld, Katsutoshi Furukawa, Aiko Ishiki, Nobuyuki Okamura, Peter Bartenstein, Marianne Dieterich, Kai Bötzel, Adrian Danek, Axel Rominger, Johannes Levin

**Affiliations:** ^1^Department of Neurology, Ludwig-Maximilians-Universität München, Munich, Germany; ^2^Department of Nuclear Medicine, University Hospital LMU Munich, Munich, Germany; ^3^German Center for Neurodegenerative Diseases (DZNE), Munich, Germany; ^4^Department of Neurology, Technical University of Munich, Munich, Germany; ^5^Institute of Clinical Neuroimmunology, Biomedical Center and University Hospital, Ludwig-Maximilians-Universität München, Munich, Germany; ^6^Institute for Clinical Radiology, Ludwig-Maximilians-Universität München, Munich, Germany; ^7^Division of Community Medicine, Tohoku Medical and Pharmaceutical University, Sendai, Japan; ^8^Department of Geriatrics and Gerontology, Institute of Development, Aging and Cancer, Tohoku University, Sendai, Japan; ^9^Division of Pharmacology, Faculty of Medicine, Tohoku Medical and Pharmaceutical University, Sendai, Japan; ^10^Munich Cluster of Systems Neurology (SyNergy), Munich, Germany; ^11^Department of Nuclear Medicine, Bern University Hospital, Bern, Switzerland

**Keywords:** tau-PET, [^18^F]-THK5351, MAO-B, astrogliosis, parkinsonian syndromes

## Abstract

Neurodegenerative parkinsonian syndromes comprise a number of disorders that are characterized by similar clinical features but are separated on the basis of different pathologies, i.e., aggregates of α-synuclein or tau protein. Due to the overlap of signs and symptoms a precise differentiation is often difficult, especially early in the disease course. Enormous efforts have been taken to develop tau-selective PET imaging agents, but strong off-target binding to monoamine oxidase B (MAO-B) has been observed across first generation ligands. Nonetheless, astrogliosis-related MAO-B elevation is a common histopathological known feature of all parkinsonian syndromes and might be itself an interesting imaging target. Therefore, this study aimed to investigate the performance of [^18^F]-THK5351, a combined MAO-B and tau tracer for differential diagnosis of parkinsonian syndromes. [^18^F]-THK5351 PET was performed in 34 patients: six with Parkinson’s disease (PD), nine with multiple system atrophy with predominant parkinsonism (MSA-P), six with MSA with predominant cerebellar ataxia (MSA-C), and 13 with progressive supranuclear palsy (PSP) Richardson’s syndrome. Volume-of-interest-based quantification of standardized-uptake-values was conducted in different parkinsonian syndrome-related target regions. PET results were subjected to multinomial logistic regression to create a prediction model discriminating among groups. Furthermore, we correlated tracer uptake with clinical findings. Elevated [^18^F]-THK5351 uptake in midbrain and diencephalon differentiated PSP patients from PD and MSA-C. MSA-C patients were distinguishable by high tracer uptake in the pons and cerebellar deep white matter when compared to PSP and PD patients, whereas MSA-P patients tended to show higher tracer uptake in the lentiform nucleus. A multinomial logistic regression classified 33/34 patients into the correct clinical diagnosis group. Tracer uptake in the pons, cerebellar deep white matter, and striatum was closely associated with the presence of cerebellar and parkinsonian symptoms of MSA patients. The current study demonstrates that combined MAO-B and tau binding of THK5351 facilitates differential diagnosis of parkinsonian syndromes. Furthermore, our data indicate a correlation of MSA phenotype with [^18^F]-THK5351 uptake in certain brain regions, illustrating their relevance for the emergence of clinical symptoms and underlining the potential of THK5351 PET as a biomarker that correlates with pathological changes as well as with disease stage.

## Introduction

Neurodegenerative parkinsonian syndromes comprise a number of disorders that are grouped together due to similar clinical features but are separated on the basis of different pathologies. Depending on the type of abnormal protein depositions, these disorders can histologically be divided into two groups: synucleinopathies and tauopathies (Dickson, 2012). Parkinson’s disease (PD) for example is characterized by intraneuronal α-synuclein aggregates (Lewy bodies) (Dickson, 2018), whereas the pathological hallmark of multiple system atrophy (MSA) are α-synuclein positive oligodendroglial cytoplasmic inclusions. A-Synuclein aggregates in MSA can be observed to a lesser extent in the form of glial nuclear inclusions, neuronal cytoplasmic inclusions, and neuronal nuclear inclusions (Papp and Lantos, 1992; Valera and Masliah, 2017). Progressive supranuclear palsy (PSP) on the other hand is defined by deposition of fibrillary aggregates of 4R tau-protein in neurons and glial cells (Dickson et al., 2010).

Especially in the early course of the disease, a precise differentiation of parkinsonian syndromes is often difficult based on clinical features alone and misdiagnosis is a frequent problem, with the most common misdiagnosis of PSP being PD and MSA (Osaki et al., 2004). This is mainly due to clinical heterogeneity and phenotypic overlap (McFarland, 2016). To this day the definite diagnosis of neurodegenerative parkinsonian syndromes still relies on post-mortem histological detection of the underlying pathology.

Recent research in positron-emission-tomography (PET) has focused on the development of tau-selective imaging agents that allow regional quantification of tau burden. While molecular imaging of tau deposits by PET initially focused on Alzheimer’s disease, the most common tauopathy, an increasing number of post-mortem and human studies are being conducted in 4R-tau positive parkinsonian syndromes (Coakeley and Strafella, 2017; Schonhaut et al., 2017). However, substantial off-target binding has been observed consistently, although with varying intensity, across most first generation tau radioligands (Brendel et al., 2017; Saint-Aubert et al., 2017; Lemoine et al., 2018; Vermeiren et al., 2018). Off-target binding to monoamine oxidase B (MAO-B), which can be elevated due to concomitant neuroinflammation and astrogliosis (Ekblom et al., 1993; Rodriguez-Vieitez et al., 2016), has recently been shown for [^18^F]-THK5351 (Ng et al., 2017; Harada et al., 2018). Therefore, [^18^F]-THK5351 may be a suitable biomarker that does not specifically indicate tau pathology but the localization of pathological and neurodegenerative processes. Indeed, recent studies have shown encouraging results regarding the use of [^18^F]-THK5351 for the differential diagnosis of PSP vs. HC and Alzheimer’s disease (Brendel et al., 2017; Ishiki et al., 2017). A significantly higher [^18^F]-THK5351 tracer retention could be detected in the midbrain and globus pallidus of PSP patients.

The aim of this study was to investigate the potential of combined MAO-B and tau binding of [^18^F]-THK5351 as a tool for differential diagnosis of neurodegenerative parkinsonian syndromes, i.e., PD, MSA, and PSP, *in vivo*. Furthermore, we sought to correlate individual regional tracer uptake with clinical phenotype and to compare PET results with magnetic resonance imaging.

## Materials and Methods

### Clinical Evaluation

A total of 34 patients from the outpatient clinic for neurodegenerative diseases at the Departments of Neurology, Ludwig-Maximilians-Universität München and Technical University of Munich, Munich, Germany, were enrolled in the study: 6 have been diagnosed with PD, 9 with MSA with predominant parkinsonism (MSA-P), 6 with MSA with predominant cerebellar ataxia (MSA-C), and 13 with PSP-Richardson’s syndrome. The intake of MAO-B inhibitors was excluded by taking the patients medication history. Diagnosis was made according to current international consensus criteria (Gilman et al., 2008; Postuma et al., 2015; Hoglinger et al., 2017). PET results from 11/13 PSP patients have previously been published (Brendel et al., 2017). Tight clinical follow-up after initial diagnosis ensured the correctness of clinical diagnosis (duration 23.4 ± 14.1 months, frequency every 3.9 ± 2.2 months). No adjustment of diagnosis was performed due conflicting PET results. Disease severity was measured with Hoehn and Yahr stage (H&Y). Furthermore, functional independence was measured using the Schwab and England Activities of Daily Living Scale (SEADL) and disease duration was recorded. The cognitive state was assessed by the Mini-Mental State Examination (MMSE). Written informed consent was obtained by all participants in accordance with the Declaration of Helsinki. Retrospective analysis of data had been approved by the local ethics committee. Demographic features of participants are listed in Table 1.

**TABLE 1:** Demographic and clinical data of the study sample.

	**PD (*n* = 6)**	**MSA-P (*n* = 9)**	**MSA-C (*n* = 6)**	**PSP (*n* = 13)**	***p***
Gender (m/f)	2/4	5/4	5/1	5/8	0.247
Age (y)	65.7 ± 8.1	68.1 ± 9.7	63.0 ± 9.1	69.2 ± 7.2	0.437
Education (y)	12.0 ± 3.8	11.8 ± 1.7	11.4 ± 0.9	12.6 ± 2.8	0.817
Disease duration (mo)	32.5 ± 30.3	44.4 ± 21.2	65.3 ± 69.3	33.7 ± 19.5	0.385
MMSE	29.4 ± 1.3	25.7 ± 4.2	28.3 ± 2.4	26.9 ± 1.5	0.072
H&Y	2.0 ± 1.5	2.8 ± 1.0	2.8 ± 1.5	3.4 ± 0.9	0.047
SEADL	76.7 ± 23.4	62.2 ± 22.8	65.0 ± 25.9	67.7 ± 16.4	0.496

*For group comparisons a Kruskal–Wallis test and *post hoc* Bonferroni-corrected Mann–Whitney tests were performed. Chi-square analysis was used to test for differences in gender distribution. PD, Parkinson’s disease; MSA-P, multiple system atrophy with predominant parkinsonism; MSA-C, multiple system atrophy with dominant cerebellar features; PSP, progressive supranuclear palsy; m, male; f, female; y, years; mo, months; MMSE, Mini-Mental State Examination; H&Y, Hoehn and Yahr Stage; SEADL, Schwab and England Activities of Daily Living scale.*

### Generation of the UMSARS-P/C Score

MSA patients can present with a combination of both parkinsonian and cerebellar features. As clinical phenotypes have been shown to be associated with predominant neurodegeneration and glial cytoplasmic inclusion pathology in the striatonigral and olivopontocerebellar system, respectively (Ozawa et al., 2004; Jellinger, 2014) we hypothesized that [^18^F]-THK5351 tracer retention in these brain areas may correlate to the amount of parkinsonian and cerebellar features.

The unified MSA rating scale (UMSARS) is the most commonly used disease-specific rating instrument in MSA and was developed to rate functional impairment independent of the underlying motor disorder (Wenning et al., 2004). To assess the amount of parkinsonian and cerebellar symptoms in our MSA cohort we calculated from the UMSARS part II, which was available in nine MSA patients, a new score (UMSARS parkinsonism/cerebellar features, i.e., UMSARS-P/C). To obtain a linear scale for correlation analysis the items facial expression, tremor at rest and increased tone referring to specific parkinsonian symptoms were scaled from 0 (no impairment) to −4 (severe impairment) whereas the items ocular motor dysfunction, action tremor, and heel–knee–shin test referring to specific cerebellar symptoms were scaled from 0 (no impairment) to +4 (severe impairment). All other items from the UMSARS part II were not included in the generation of the UMSARS-P/C score, as they measure functional impairment of complex movements and can be caused by both parkinsonian and cerebellar symptoms. Therefore, the UMSARS-P/C potentially ranges from −12 representing severe pure parkinsonism to +12 representing severe pure cerebellar symptoms. As most MSA patients present with a combination of parkinsonian and cerebellar features the score ranged from −4 to +4 in our study cohort.

### PET Imaging

Automated production of [^18^F]-THK5351 was performed on a Raytest^®^ SynChrom R&D single reactor synthesizer as reported previously (Brendel et al., 2017).

All emission recordings were performed in a previously established protocol (Brendel et al., 2017). In brief, images were acquired using a GE Discovery 690 PET/CT scanner. A prior low-dose CT scan was performed for attenuation correction. Dynamic three-dimensional emission recordings were acquired during an interval of 50–70 min after intravenous injection of 183 ± 4 MBq [^18^F]-THK5351.

The PNEURO data processing pipeline of PMOD Version 3.5 (PMOD Technologies Ltd., Zurich, Switzerland) was used for spatial normalization of all [^18^F]-THK5351 images to the Montreal Neurological Institute (MNI) space. All individual PET images were spatially normalized to the previously established [^18^F]-THK5351 template using the PMOD FUSION tool (equal modality; non-linear warping; 16 iterations; frequency cutoff 3; regularization 1.0; no thresholding; 8 mm transient input smoothing). Global mean intensity scaling was used for image normalization.

For semiquantitative analyses, volume of interest (VOIs) were predefined in target regions known to be affected by MAO-B elevation and tau pathology in parkinsonian syndromes: midbrain, diencephalon, striatum, nucleus lentiformis, pons, and cerebellar deep white matter. Mean standard-uptake-value ratios (SUVR) relative to the global mean (SUVRGLM) were calculated for each VOI in all subjects.

### Magnetic Resonance Imaging

Magnetic resonance imaging (MRI) data were available in 26/34 patients. The remaining patients either refused MRI or had a contraindication to MRI, e.g., permanent cardiac pacemakers.

In accordance with prior studies assessing morphometric MRI parameters for differential diagnosis of parkinsonism (Cosottini et al., 2007; Heim et al., 2017), we determined the ratio of the midbrain cross-sectional area scaled by the pons area. All measurements were performed in Horos open source medical image viewer^1^ on mid-sagittal images of T1/T2w sequences. A midsagittal plane of the brain volume passing through the middle of the interpeduncular fossa, the middle of the aqueduct, and the falx cerebri was chosen (each determined on axial images). The midbrain area was determined by tracing the contour of the midbrain down to a line parallel to the hypothetic conjunction between the genu and splenium of the corpus callosum touching the superior part of the pons. For the determination of the pons midsagittal area, we used the region underneath, extending from the lower bound of the midbrain area down to a parallel line touching the inferior border of the pons.

### Statistical Analysis

Data were analyzed using IBM SPSS Statistics for Windows (Version 25.0., IBM Corp., Armonk, NY, United States). Non-dichotomized mean scores of demographic and clinical data were compared across the four groups (PD, MSA-P, MSA-C, and PSP patients) via Kruskal–Wallis test and *post hoc* Bonferroni corrected Mann–Whitney tests. Chi-square analysis was used to test for differences in gender distribution across all groups. Standard statistical significance level was set at *p* < 0.05.

For group comparisons of semiquantitative PET results in different target VOIs a Kruskal–Wallis test was performed. Significance levels for the Kruskal–Wallis test were adjusted according to Bonferroni correction (*p* < 0.0083). Results of *post hoc* tests were regarded significant if they survived an additional Bonferroni correction for multiple pairwise comparisons. Spearman’s test was used to explore significant correlations between PET results and clinical parameters (H&Y, SEADL, disease duration, UMSARS-P/C) and the midbrain-to-pons area ratio calculated from MRI, respectively.

Four-category multinomial logistic regression was used to classify among the four disease groups using significant semiquantitative PET results as predictor variables. Additionally, multinomial logistic regression using MRI midbrain-to-pons area ratio as predictor was performed to compare the diagnostic utility of [^18^F]-THK5351 PET and MRI.

## Results

### Demographic and Clinical Data

Demographics and clinical scores of the study sample are provided in Table 1. Patient groups did not differ significantly in terms of age, gender, education, disease duration, MMSE, and SEADL. Kruskal–Wallis test detected significant group differences of H&Y stage with lowest scores in PD and highest scores in PSP.

### Regional PET Analyses

**FIGURE 1:**
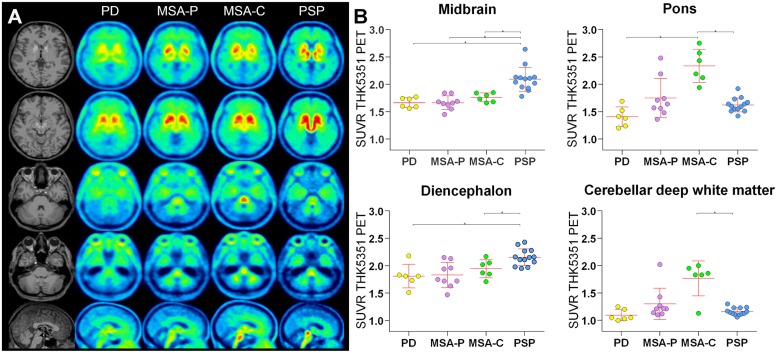
[^18^F]-THK5351 tracer uptake. **(A)** High midbrain uptake in axial and sagittal slices allows visual discrimination of the PSP patient group from other patient groups. Significantly higher [^18^F]-THK5351 uptake in the diencephalon was detected in PSP patients compared to PD and MSA-C patients. In contrast, in MSA-C patients, but not in MSA-P patients, [^18^F]-THK5351 uptake was especially high in the pons and cerebellar deep white matter. **(B)** Tracer uptake in the midbrain, pons, diencephalon, and cerebellar deep white matter. ^∗^ indicates significant differences.

Significant differences of [^18^F]-THK5351 uptake were observed in the diencephalon, midbrain, pons, and cerebellar deep white matter (Kruskal–Wallis test, Table 2 and Figures 1A,B). [^18^F]-THK5351 uptake in the lentiform nucleus was highest in MSA-P patients but did not survive the significance threshold for multiple comparisons. No significant differences could be detected for the striatum.

**TABLE 2:** [^18^F]-THK5351 uptake.

	**PD**	**MSA-P**	**MSA-C**	**PSP**	***p***
Striatum	2.24 ± 0.26	2.38 ± 0.21	2.31 ± 0.15	2.47 ± 0.16	0.101
Lentiform nucleus	1.83 ± 0.17	2.25 ± 0.38	2.05 ± 0.36	2.00 ± 0.11	0.042
Diencephalon	1.81 ± 0.22^d^	1.88 ± 0.25	1.87 ± 0.14^d^	2.16 ± 0.15^a,c^	0.005
Midbrain	1.66 ± 0.09^d^	1.66 ± 0.13^d^	1.76 ± 0.08^d^	2.09 ± 0.22^a,b,c^	<10^−4^
Pons	1.41 ± 0.18^c^	1.75 ± 0.36	2.33 ± 0.30^a,d^	1.62 ± 0.13^*c*^	0.001
Cerebellar deep white matter	1.10 ± 0.10	1.30 ± 0.29	1.77 ± 0.32^d^	1.16 ± 0.07^c^	0.006

*[^18^F]-THK5351 uptake: Mean standard-uptake-value ratios relative to the global mean. For group comparisons a Kruskal–Wallis test and *post hoc* Mann–Whitney tests were performed. Significance levels of both tests were adjusted according to Bonferroni correction (*p* < 0.0083). PD, Parkinson’s disease; MSA-P, multiple system atrophy with predominant parkinsonism; MSA-C, multiple system atrophy with dominant cerebellar features; PSP, progressive supranuclear palsy. Significantly different compared to ^*a*^PD, ^*b*^MSA-P, ^*c*^MSA-C, and ^*d*^PSP.*

*Post hoc* Bonferroni tests showed that PSP patients had higher [^18^F]-THK5351 uptake in the diencephalon compared to PD and MSA-C and had higher [^18^F]-THK5351 uptake in the midbrain compared to all other patient groups whereas MSA-C patients had higher [^18^F]-THK5351 uptake in the pons compared to PSP and PD patients and had higher [^18^F]-THK5351 uptake in cerebellar deep white matter compared to PSP patients (Figures 1A,B).

### Logistic Regression

The multinomial logistic regression model was able to accurately classify 33/34 (97.1%) patients using the [^18^F]-THK5351 uptake in the diencephalon, midbrain, pons, and cerebellar deep white matter as predictor variables (*p* < 0.05, *R*^2^ = 0.98). A more conservative and therefore potentially clinically more meaningful regression model using only the [^18^F]-THK5351 uptake in the midbrain and pons as predictor variables was still able to correctly classify 29/34 (85.3%) patients (*p* < 0.05, *R*^2^ = 0.93). A model using the MRI midbrain-to-pons area ratio as predictor, however, was able to accurately classify 17/26 (65.4%) patients (*p* < 0.05, *R*^2^ = 0.68).

### Correlation Analysis

In order to assess the potential of [^18^F]-THK5351 as a biomarker of disease stage and progression, a correlation analysis of tracer uptake and clinical parameters as well as with MRI atrophy was conducted. We observed a significant positive correlation of H&Y stage with [^18^F]-THK5351 uptake in the lentiform nucleus (*r*_s_ = 0.38, *p* = 0.03), the midbrain (*r*_s_ = 0.53, *p* = 0.001), and the diencephalon (*r*_s_ = 0.42, *p* = 0.01) and a negative correlation of the SEADL with [^18^F]-THK5351 uptake in the lentiform nucleus (*r*_s_ = −0.49, *p* = 0.003) and pons (*r*_s_ = −0.43, *p* = 0.01). There was no significant correlation of tracer uptake with disease duration.

**FIGURE 2:**
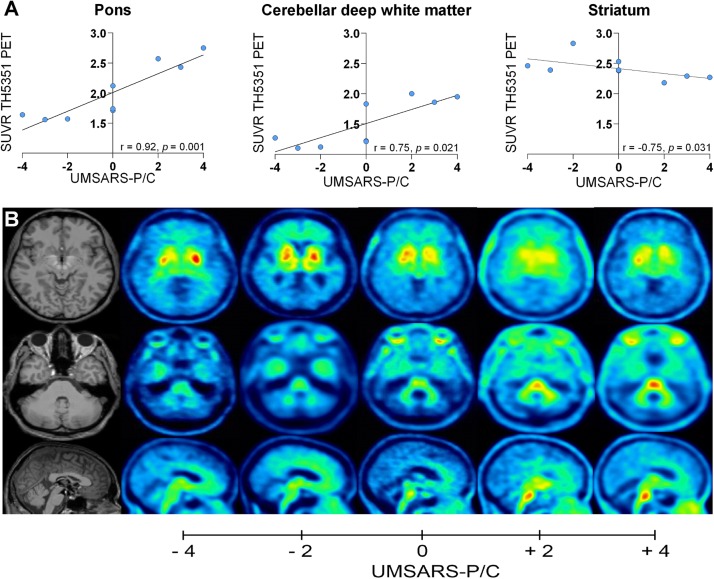
Correlation of [^18^F]-THK5351 tracer uptake with UMSARS-P/C: **(A)** Correlation of [^18^F]-THK5351 tracer uptake in the pons, cerebellar deep white matter, and striatum with UMSARS-P/C. **(B)** Axial and sagittal slices showing individual tracer uptake of patients with an UMSARS-P/C score of –4, –2, 0, +2, and +4.

As clinical variants of MSA are known to be associated with morphologic phenotypes of striatonigral and olivopontocerebellar degeneration an additional correlation analysis of regional [^18^F]-THK5351 tracer uptake and the amount of parkinsonian and cerebellar features measured by the UMSARS-P/C was performed. The UMSARS-P/C was positively correlated with tracer uptake in the pons (*r*_s_ = 0.92, *p* = 0.001) and cerebellar deep white matter (*r*_s_ = 0.75, *p* = 0.02) and negatively correlated with tracer uptake in the striatum (*r*_s_ = −0.71, *p* = 0.03) (Figures 2A,B). Figure 2B demonstrates tracer uptake of patients with an UMSARS-P/C score of −4, −2, 0, +2, and +4 in these regions.

The MRI midbrain-to-pons area ratio showed a positive correlation with [^18^F]-THK5351 uptake in the pons (*r*_s_ = 0.49, *p* = 0.01) and cerebellar deep white matter (*r*_s_ = 0.47, *p* = 0.02) and a negative correlation with tracer uptake in the diencephalon (*r*_s_ = −0.54, *p* = 0.01) and midbrain (*r*_s_ = −0.42, *p* = 0.03).

## Discussion

We present the first study demonstrating the value of a combined MAO-B and tau radioligand for the differential diagnosis of neurodegenerative parkinsonian syndromes including primary tauopathies (PSP) and α-synucleinopathies (PD, MSA). Tracer uptake levels of [^18^F]-THK5351 in the diencephalon, midbrain, pons, and cerebellar deep white matter are significant predictors of clinical diagnosis. Furthermore, our data indicate a strong association between region-specific tracer uptake and the amount of parkinsonian and cerebellar symptoms in MSA.

In recent years, enormous efforts have been taken to develop tau-selective PET imaging agents (Xia et al., 2013; Okamura et al., 2014). First generation tau PET tracers like [^18^F]-AV1451 and [^18^F]-THK5351 have shown in PSP patients high tracer uptake in regions that are known from histological examination post mortem to be severely affected by tau pathology in PSP (Williams et al., 2007; Dickson et al., 2010). However, substantial off-target binding has been observed for all first generation tau-tracers. [^18^F]-AV1451 for example shows significant off-target binding to neuromelanin-containing cells from the substantia nigra, vascular structures like the choroid plexus and dural venous sinuses, and the basal ganglia in general (Marquie et al., 2015; Lowe et al., 2016) whereas [^18^F]-THK5351 has been shown to bind to MAO-B. In a recent human blocking study a single oral dose of 5 mg of selegiline has led to a reduction of [^18^F]-THK5351 retention of up to 51.8% (Ng et al., 2017). Furthermore, imaging-pathology studies of autopsy-confirmed patients who underwent [^18^F]-THK5351 PET before death showed a significant correlation between *in vivo* [^18^F]-THK5351 uptake and post mortem MAO-B levels (Harada et al., 2018; Ishiki et al., 2018). These results suggest a high amount of tracer retention being due to binding to MAO-B.

MAO-B catalyzes the oxidative deamination of biogenic amines like dopamine. As MAO-B is mainly located in the mitochondrial outer membrane of astrocytes (Shih et al., 1999), especially reactive astrocytes, it has been proposed as a marker of astrogliosis (Ekblom et al., 1993; Rodriguez-Vieitez et al., 2016). A recent post mortem study in parkinsonian conditions showed elevated MAO-B levels in the putamen and midbrain of MSA patients, elevated MAO-B levels in the caudate nucleus, putamen, and midbrain of PSP patients but no elevation of MAO-B in the basal ganglia of PD patients (Tong et al., 2017). Furthermore, MAO-B was positively correlated to astrocyte proteins like vimentin, HSP27, and glial fibrillary acid protein. Hence, MAO-B in the atypical parkinsonian syndromes MSA and PSP was significantly increased in brain areas affected by neurodegeneration.

MAO-B off target binding brought [^18^F]-THK5351 into disrepute during the last 2 years. Nonetheless, the opportunity to assess both astrogliosis and tau pathology by a single *in vivo* examination has several major advantages as proven by the current data: First, neuropathological features of both tauopathies (astrogliosis and tau) and α-synucleinopathies (astrogliosis) can be assessed by a positive contrast. In particular a pure tau tracer would not be able to detect MSA or PD patients, whereas a pure α-synuclein ligand would blind to PSP. Second, the varying topology of neuropathology allows to discriminate clinically similar parkinsonian syndromes with different underlying proteinopathies. Third, astrogliosis has been shown to be an early biomarker in the time course of Alzheimer’s disease (Rodriguez-Vieitez et al., 2016; Rodriguez-Vieitez and Nordberg, 2018) which might also be the case for parkinsonian syndromes, allowing an early discrimination.

As expected from our previous comparison of PSP patients against healthy controls (Brendel et al., 2017), a significantly higher [^18^F]-THK5351 uptake in the midbrain of PSP patients compared to all other patient groups was observed, a region that is known to be affected by both increased MAO-B levels (Tong et al., 2017) and tau pathology (Dickson et al., 2010) in PSP. In the diencephalon, a region in which tau-pathology in PSP is also abundant (Williams et al., 2007; Dickson et al., 2010), a significantly higher tracer uptake could be detected in PSP compared to PD and MSA-C, while tracer uptake also showed a trend toward significance in comparison to MSA-P (*p* = 0.015). In contrast, no significant group differences concerning tracer uptake could be detected in the striatum. In the lentiform nucleus, that is composed of putamen and globus pallidus, tracer uptake was highest in MSA-P patients, followed by MSA-C and PSP patients, but did not survive the significance threshold for multiple comparisons. This is probably due to the combination of elevated MAO-B levels and tau pathology in the putamen of PSP patients and the fact that MAO-B levels have been shown to be highly increased in MSA patients (Tong et al., 2017). Tracer uptake in the lentiform nucleus was lowest in the PD patient group which is in good agreement with the fact that MAO-B levels have been shown to be normal and astrogliosis to be limited in the basal ganglia of PD patients (Song et al., 2009).

In MSA-C pontine [^18^F]-THK5351 uptake was significantly elevated when compared to PSP and PD patients and was also elevated when compared to MSA-P but did not survive the significance threshold for multiple comparisons (*p* = 0.013). Furthermore, tracer uptake in the cerebellar deep white matter of MSA-C patients was increased in comparison to PSP patients and showed a trend toward significance in comparison to PD (*p* = 0.010) and MSA-P (*p* = 0.077) patients. Both regions are known to be early foci of α-synuclein pathology in MSA-C (Brettschneider et al., 2017). Recent studies have shown that astrogliosis in MSA and thereby increased MAO-B levels correlate to the presence of oligodendrocytes with α-synuclein glial cytoplasmic inclusions (Radford et al., 2015). This fact suggests that localized presence of α-synuclein underlies astrocytic pathology in MSA and explains the high amount of tracer uptake detected in our MSA-C patient group in these regions.

Due to the overlap of signs and symptoms of parkinsonian syndromes a reliable diagnosis remains a major clinical challenge. However, diagnostic accuracy is essential for estimation of prognosis, optimizing patient care, and allocation to therapeutic trials. While the accuracy of PD diagnosis in movement disorders centers has been shown to be almost 99%, the diagnostic accuracy of atypical parkinsonian syndromes is limited being only 77% (Hughes et al., 2002). When the diagnosis is made by general neurologists diagnostic accuracy is even worse being 75 and 61%, respectively (Joutsa et al., 2014). Imaging modalities may help to increase diagnostic certainty. In our current study the optimal multinomial logistic regression model was able to accurately classify 33/34 patients using [^18^F]-THK5351 uptake in the diencephalon, midbrain, pons, and cerebellar deep white matter as predictor variables. A more conservative and therefore potentially clinically more meaningful model using only tracer uptake in the midbrain and pons as predictors was still able to classify 85.3% of patients correctly. Previous studies have shown good diagnostic accuracy of MRI midbrain-to-pons area ratio for differentiating patients with PSP from patients with MSA and PD (Cosottini et al., 2007; Moller et al., 2017). A multinomial logistic regression model using the midbrain-to-pons area ratio as predictor variable, however, only led to a classification accuracy of 65.4% and was therefore worse than the model using semiquantitative PET results as predictors. Our data therefore provide evidence for [^18^F]-THK5351 uptake in the diencephalon, midbrain, pons, and cerebellar deep white matter to be of high predictive value in diagnosing the four parkinsonian syndromes under investigation. Combined MAO-B and tau binding of [^18^F]-THK5351 seems to be of value for differentiation of parkinsonian syndromes and may be a useful diagnostic tool in the absence of 4R-tau- and α-synuclein-selective ligands.

In order to evaluate therapeutic agents, biomarkers that correlate with pathological changes as well as with clinical phenotype are urgently needed. To be clinically relevant the respective biomarker must therefore show a good correlation with disease stage and progression and may then be used for the evaluation of therapeutic responses (Frank and Hargreaves, 2003). Recent studies, including our group, have detected a significant correlation between the intensity of [^18^F]-THK5351 and [^18^F]-AV1451 tracer uptake in the midbrain and disease severity measured by the PSP Rating Scale in PSP patients (Brendel et al., 2017; Whitwell et al., 2017). In our MSA cohort tracer uptake in the striatum, pons, and cerebellar deep white matter correlated with the amount of parkinsonian and cerebellar symptoms, respectively. This finding illustrates the clinical relevance of the striatum for the emergence of parkinsonian symptoms and the relevance of the pons and cerebellar deep white matter for the emergence of cerebellar symptoms in MSA and underlines the potential of [^18^F]-THK5351 PET as a biomarker assessing phenotype and disease stage.

Furthermore, our data disclosed a positive/negative correlation between midbrain-to-pons area ratio calculated from MRI with [^18^F]-THK5351 uptake in the pons and cerebellar deep white matter as well as with tracer uptake in the diencephalon and midbrain. This correlation is driven by the combined effect of pontine atrophy in MSA-C patients that show a high amount of tracer uptake in the pons and cerebellar deep white matter and midbrain atrophy in PSP patients that have significantly higher tracer uptake in the diencephalon and midbrain. Therefore, [^18^F]-THK5351 retention seems to be closely related to other markers of neuronal injury. This finding suggests that the presence of astrogliosis-related MAO-B elevation and tau, respectively, strongly affects neurodegeneration and assures us about well-capturing neuropathological changes.

A limitation of the current study that needs to be considered is the absence of a neuropathological confirmation of diagnosis which reflects the moderate clinical severity within our patients, most of whom are alive at the time being. However, the lack of a neuropathological confirmation is a general problem in studies investigating neurodegenerative disorders at an early disease stage. Therefore, there remains the possibility that some cases had a mismatch of clinical diagnosis and underlying pathology. However, the present results have face validity given the agreement between [^18^F]-THK5351 uptake and the known topology of tau distribution in PSP patients and the topology of astrogliosis in the patient groups under investigation, respectively. Furthermore, the tight clinical follow-up over 23.4 ± 14.1 months ensured us about the clinical diagnosis. Furthermore, a more deep and specific cognitive assessment would have been desirable. However, as the current study is retrospective by nature, this was not feasible. We further acknowledge the small sample size, especially of MSA-C patients. However, it should be noted that the current study is the first investigating [^18^F]-THK5351 uptake in MSA. Nonetheless a larger multicenter investigation of this rare disease will be needed to evaluate the utility of [^18^F]-THK5351 as a combined radiotracer of tau and astrogliosis in neurodegenerative diseases.

Keeping these limitations in mind our findings reveal combined tau and MAO-B binding of [^18^F]-THK5351 to facilitate differential diagnosis of neurodegenerative parkinsonian syndromes and to correlate with phenotype and MRI atrophy. [^18^F]-THK5351 may have limited utility as a specific biomarker of tau but may be a promising marker of neuroinflammation accompanying neurodegeneration.

## Data Availability

The datasets generated for this study are available on request to the corresponding author.

## Ethics Statement

Written informed consent was obtained by all participants in accordance with the Declaration of Helsinki. Retrospective analysis of data had been approved by the Local Ethics Committee.

## Author Contributions

SoS and MB: research project – conception, organization, and execution; statistical analysis – design and execution; and manuscript preparation – writing of the first draft. CP, LB, ES, B-SR, JS, GR, and StS: research project – execution and manuscript preparation – review and critique. GH, PB, and MD: statistical analysis and manuscript preparation – review and critique. KF, AI, NO, KB, and AD: research project – organization and manuscript preparation – review and critique. AR and JL: research project – conception and organization and statistical analysis and manuscript preparation – review and critique.

## Conflict of Interest Statement

GH has served on the advisory boards for AbbVie, Alzprotect, Asceneuron, Biogen, Novartis, Roche, Sanofi, UCB; has received honoraria for scientific presentations from Abbvie, Biogen, Roche, Teva, UCB; has received research support from CurePSP, the German Academic Exchange Service (DAAD), German Parkinson’s Disease Foundation (DPG), German PSP Association (PSP Gesellschaft), German Research Foundation (DFG) and the German Ministry of Education and Research (BMBF), International Parkinson’s Fonds (IPF); and has received institutional support from the German Center for Neurodegenerative Diseases (DZNE). The remaining authors declare that the research was conducted in the absence of any commercial or financial relationships that could be construed as a potential conflict of interest.

## Footnotes

1 http://horosproject.org/

## Abbreviations

AUC: area under the curve
H&Y: Hoehn and Yahr stage
MAO-B: monoamine oxidase B
MMSE: Mini-Mental State Examination
MSA: multiple system atrophy
MSA-C: MSA with predominant cerebellar ataxia
MSA-P: MSA with predominant parkinsonism
PD: Parkinson’s disease
PSP: progressive supranuclear palsy
ROC: receiver operating characteristic
SEADL: Schwab and England Activities of Daily Living scale
SUVR: standard-uptake-value ratio
UMSARS: unified MSA Rating Scale
UMSARS-P/C: UMSARS-parkinsonism/cerebellar features
VOI: volume-of-interest.
